# Size and surface charge characterization of nanoparticles with a salt gradient

**DOI:** 10.1038/s41467-020-15889-3

**Published:** 2020-05-11

**Authors:** Martin K. Rasmussen, Jonas N. Pedersen, Rodolphe Marie

**Affiliations:** Department of Health Technology, Technical University of Denmark, Oersteds Plads building 345c, 2800 Kongens Lyngby, Denmark

**Keywords:** Nanoparticles, Nanofluidics

## Abstract

Exosomes are nanometer-sized lipid vesicles present in liquid biopsies and used as biomarkers for several diseases including cancer, Alzheimer’s, and central nervous system diseases. Purification and subsequent size and surface characterization are essential to exosome-based diagnostics. Sample purification is, however, time consuming and potentially damaging, and no current method gives the size and zeta potential from a single measurement. Here, we concentrate exosomes from a dilute solution and measure their size and zeta potential in a one-step measurement with a salt gradient in a capillary channel. The salt gradient causes oppositely directed particle and fluid transport that trap particles. Within minutes, the particle concentration increases more than two orders of magnitude. A fit to the spatial distribution of a single or an ensemble of exosomes returns both their size and surface charge. Our method is applicable for other types of nanoparticles. The capillary is fabricated in a low-cost polymer device.

## Abstract (web summary)

Exosomes are used as disease biomarkers, but their characterization in biological samples is challenging. Here the authors achieve simultaneous characterization of size and zeta potential of individual nanoparticles and particle mixtures at physiological salinity conditions, exploiting a salt gradient in a capillary channel.

## Introduction

Purification and analysis of nanoparticles, as routinely performed when isolating exosomes from liquid biopsies, are often based on size and surface properties, for example, their biochemical components or zeta potential^1^. The same parameters are also important for the synthesis and functionality of both solid state and soft matter synthetic colloids. These can, for example, be designed to promote interactions with living cells, have active reaction sites on their surfaces, or show specific optical properties.

The zeta potential, which depends on the surface charge, is important for the stability of nanoparticles in suspension^2^ and is also the major factor in the initial adsorption of nanoparticles onto the cell membrane^3^. After adsorption, the endocytotic uptake rate depends on the particle size^4^. The zeta potential and size thus affect nanoparticle toxicity^5^.

Exosomes, present in most body fluids, have received great interest due to their potential as biomarkers and use in precision medicine^6–8^. The zeta potentials and sizes of exosomes from various body fluids are significantly different as they interact with different cellular targets. So these parameters reflect both the origin and the endocytotic pathway that the exosomes may use^3^, and they are integral properties of the cell-to-cell signaling system. Control of the size and zeta potential are thus important factors for the effectiveness of nanoparticles for drug delivery^1,9–11^, and allows for specifying the cellular targets for, for example, liposomes^4,12,13^, gold nanoparticles^14^, and copolymer micelles^15^.

For soft matter nanoparticles, for example, exosomes or liposomes, traditional purification methods pose a number of challenges. Ultracentrifugation, one of the most commonly used concentration methods, is time consuming and suffers from contamination^1,16^. Size-based filtration through membranes can result in deformations or even break the particles^1,17^. Current characterization techniques for nanoparticles also all have challenges, for example, cryo-electron microscopy that is a destructive method^18^. Measurements of size and zeta potential are routinely performed on the same instrument by dynamic light scattering (DLS) and laser Doppler electrophoresis (LDE), but require multiple experiments, and are challenged by polydisperse samples, for example, exosomes isolated from body fluids.

The fast development within micro- and nanofluidics allows for reduced reagent consumption in lab-on-a-chip devices. Microfluidics-based purification and characterization methods have become increasingly important for nanoparticle research in general, and in particular for soft matter nanoparticles^1,19–22^. In micro- and nanofluidic devices, nanoparticles can be exposed to gradients in, for example, electric potential^23^, temperature^24^, pH^25^, and solute concentration^26^, and such gradients induce phoretic transport processes that depend on particle properties^27^. In an electric field, particles experience electrophoretic transport, which is, for example, utilized in size-dependent separation of DNA molecules in microlithographic arrays^28^, and for focusing of proteins in nanochannels^23^. In the presence of a solute gradient, particles will spontaneously migrate along the concentration gradient due to diffusiophoresis^26,29^. Diffusiophoresis offers much higher transport rates than regular diffusion and has been used for efficient filling of dead-end channels^30–32^, water filtration^33^, colloidal particle accumulation^34^, and active collective colloidal behavior^30,35,36^. For a Debye length comparable to the particle size, the diffusiophoretic velocity shows much higher particle size dependence than its electrophoretic counterpart, and it can thus be utilized for size-dependent separation of nanoparticles^30^.

The physical mechanism behind diffusiophoresis also gives rise to a diffusioosmotic fluid flow in the presence of a solute gradient near a charged plane^26^, for example, in a nanochannel^37^. This opens up for the possibility to manipulate particles by combining the diffusiophoretic migration with the diffusioosmotic flow of the surrounding fluid. The fluid velocity can be controlled efficiently by the nanochannel geometry.

In this article, we show how to combine diffusiophoretic particle transport with the oppositely directed diffusioosmotic fluid transport in order to trap, concentrate, and characterize nanoparticles in a nanofluidic device. The advantage of our method is that particles are trapped at an equilibrium position, not by a transient fluid flow^30^. This allows us to track individual particles in dilute suspensions for several minutes and to infer their properties from the trajectories. For ensembles, particle concentrations are much higher in the trap than in the initial sample, and particles are characterized based on the concentration profile. Data analysis is based on a closed set of equations without the need for calibration to simulations^19^. Our nanofluidic device does not require any electrodes to produce an applied electrical field in combination with the salt gradient^23^, and the trapping depends solely on the salt gradient and the geometry of the nanochannel. Hence, the device can be fabricated by scalable production processes, for example, polymer injection molding.

**Fig. 1:**
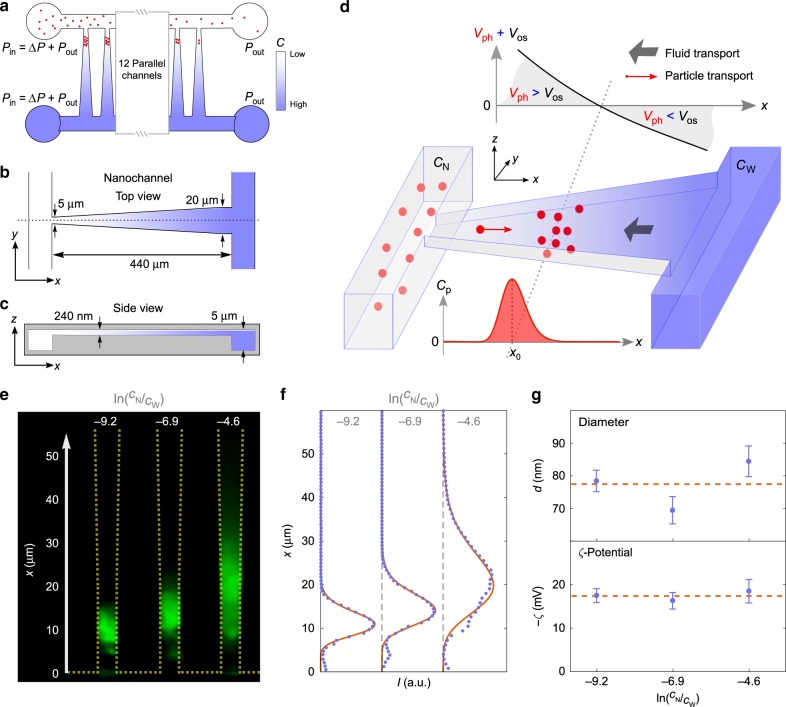
Trapping of nanoparticles in a salinity gradient. **a** Schematic of the nanofluidic device with 16 funnel-shaped, parallel nanochannels connecting two microchannels. Pressure differences Δ*P* between the in- and outlets continuously drive buffers through the microchannels. Different salt concentrations *C*_N_ and *C*_W_ in the two microchannels at the narrow and wide ends of the nanochannels, respectively, establish a salt gradient across the nanochannels. Nanoparticles (red dots) are loaded in the microchannel with low salinity and some get trapped in the nanochannels. **b**, **c** Schematic top and side view of the funnel-shaped nanochannels, respectively. **d** Schematic of nanoparticle trapping in a funnel-shaped nanochannel by diffusioosmosis and diffusiophoresis. The gradient induces a diffusioosmotic fluid flow velocity ν_os_ and a diffusiophoretic particle velocity ν_ph_. Nanoparticles (red dots) get trapped around the position *x*_0_ where the fluid and particle velocities balance each other. The concentration of nanoparticles in the nanochannel is denoted *C*_p_(*x*). **e** Composite fluorescence microscope image of trapped exosomes in the same nanochannel (outlined with yellow) for three different salinity gradients, that is, different ln(*C*_N_/*C*_W_) values. Images were averaged over 10 s. **f** Fluorescence intensity along the nanochannels for the data shown in **e** (blue points). Full, red lines are independent fits to *C*_p_(*x*) in Eq. (7). Fit parameters are the diameter *d* and the zeta potential *ζ*. **g** Exosome diameters and zeta potentials from fits shown in **f**. Red, dotted lines are the weighted averages. Error bars are the standard errors on the means for measurements in three different nanochannels (*n* = 3).

**Fig. 2:**
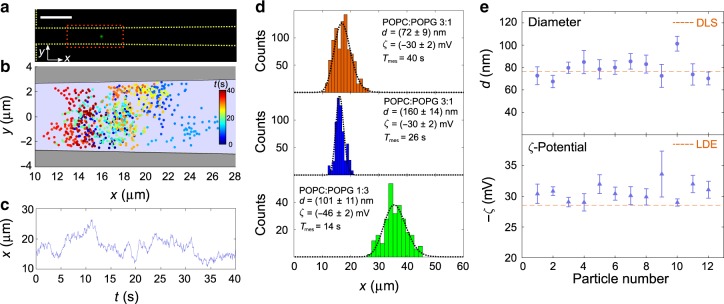
Tracking a single, trapped particle. **a** Fluorescence microscopy image of a single, trapped liposome. Salt concentrations in the microchannels are fixed at *C*_N_ equal to 10^−3^× PBS and *C*_W_ equal to 10× PBS, that is, ln(*C*_N_/*C*_W_) = −9.2. Scale bar is 10 μm. **b** Measured positions in the nanochannel for the area indicated with the red box in **a**. The particle is tracked for *T*_mes_ = 40 s and imaged at 20 frames per second. **c** *x*-Coordinates versus time. **d** Histograms and fitted distributions obtained from single-particle tracking of liposomes from three different populations with different diameters and zeta potentials. Upper panel corresponds to data in **c**. **e** Diameters and zeta potentials of individual particles from the POPC:POPG 3:1 population with *d*_DLS_ = (76 ± 3) nm and *ζ*_LDE_ = (−28 ± 1) mV. Results for particle no. 1 are from the the upper panel of **d**. Error bars are the standard deviations obtained from the fits to histograms in **d**.

**Fig. 3:**
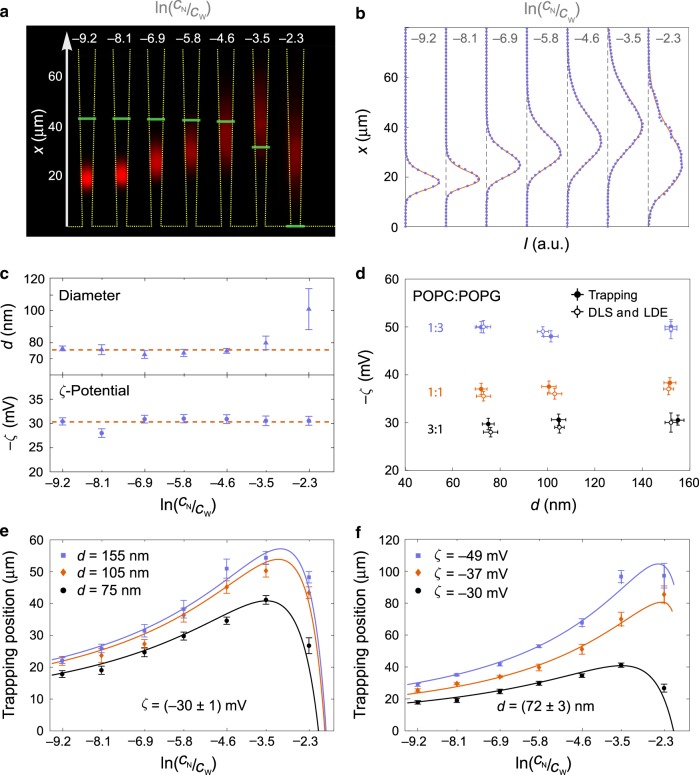
Trapping of different types of liposomes in nanochannels. **a** Composite fluorescence microscope image of trapped POPC:POPG 3:1 liposomes in nanochannels (outlined with yellow) measured at seven different salinity gradients (*C*_N_ in the range 10^−3^× PBS to 1× PBS and *C*_W_ fixed at 10× PBS). Liposomes have diameters *d*_DLS_ = (76 ± 3) nm and zeta potentials *ζ*_LDE_ = (−28 ± 1) mV. Green, horizontal lines mark the positions of physiological salinity (1× PBS). The images were averaged over 10 s. **b** Fluorescence intensity along the nanochannel (blue dots) and corresponding fits (red line). **c** Extracted liposome diameter and zeta potential of the same liposomes at different salinity gradients. Dashed, red lines are weighted averages over the fit parameters for the different salinity gradients. **d** Summary of results for diameters and zeta potentials obtained from trapping, DLS, and LDE experiments for the nine different combinations of zeta potentials and diameters. Each trapping data measurement is obtained similarly as the average values shown in **c**. **e** Trapping positions of POPC:POPG 3:1 liposome populations with three different sizes and an average zeta potential of (−30 ± 1) mV. Solid lines are the theoretical trapping positions with the corresponding values from the trapping method in **d** as input parameters and an independent calibration of the fluid flow. **f** Trapping position of liposomes with three different compositions, and, consequently, zeta potentials, but almost identical sizes [average diameter (72 ± 3) nm]. Solid lines are the theoretically predicted trapping positions calculated as in **e**. All error bars are the standard errors on the means (*n* = 3).

**Fig. 4:**
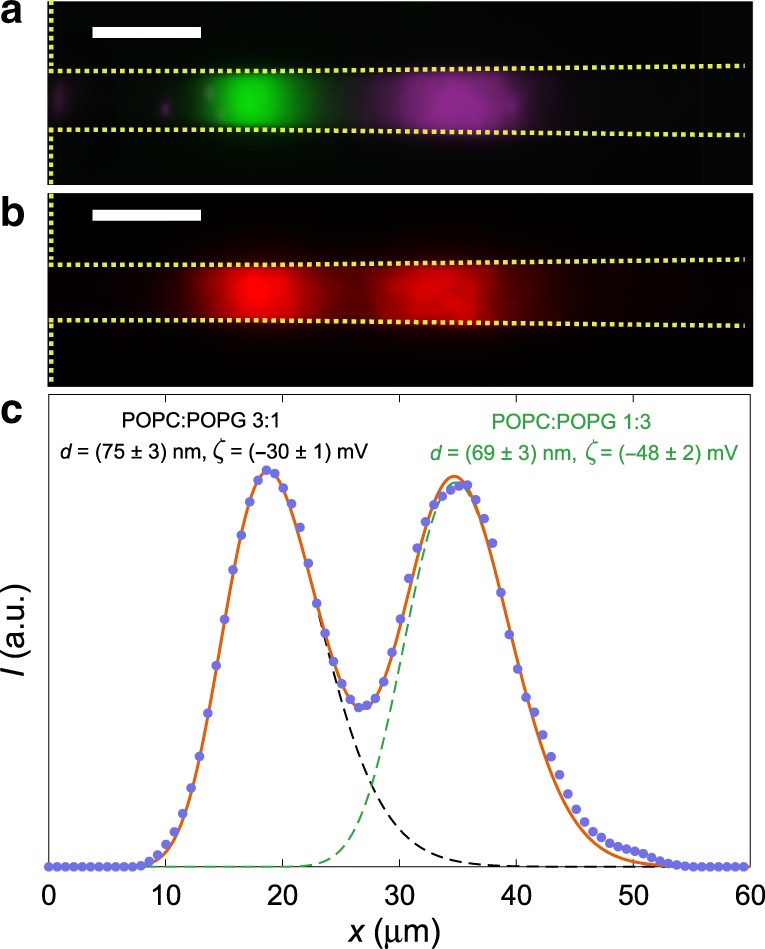
Separating a mixture of liposomes. **a** Fluorescence image of trapped POPC:POPG 3:1 and POPC:POPG 1:3 liposomes marked with different fluorophores in a nanochannel (outlined with yellow). Experiment is performed at ln(*C*_N_/*C*_W_) =− 8.1. Scale bar is 10 μm. **b** Same as **a**, but for liposomes with identical fluorophores. The image is an average over 10 s. Scale bar is 10 μm. **c** Fluorescence intensity of the two trapped populations shown in **b** (blue dots) and corresponding fit (red line) of the full distribution describing two particle populations, *C*_p_(*x*) = *w*_1_*C*_p,1_(*x*) + *w*_2_*C*_p,2_(*x*). Here *C*_p,1_ and *C*_p,2_ are the fits to Eq. (7) for the two particle populations, and *w*_1_ and *w*_2_ are weight factors with *w*_1_ + *w*_2_ = 1.

We first demonstrate the method on an ensemble of exosomes (Fig. 1). We then determine the size and surface charge of individual liposomes (Fig. 2), establishing the method as a single-particle tool. Next, we characterize ensembles of liposomes with nine different combinations of diameters and lipid compositions (Fig. 3). Finally, we perform on-chip separation and characterization of liposome populations based on size and lipid composition (Fig. 4).

## Results

### Principle of trapping by a salt gradient in a nanochannel

Our nanofluidic device traps nanoparticles in 16 parallel funnel-shaped nanochannels bridging two microchannels, see Fig. 1a–c. Different salt concentrations in the two microchannels maintain a position-dependent salt concentration *C*(*x*) across each nanochannel. The salt concentrations are identical in all nanochannels, that is, there are 16 parallel experiments on the device. The salt gradients in the nanochannels cause a diffusiophoretic particle migration^26^ and an oppositely directed diffusioosmotic fluid flow^37^, and they both depend on the relative, local salt gradient $C^{\prime}\left( x \right)/C\left( x \right) = \partial _x{\mathrm{ln}}\,C\left( x \right)$. Particles are trapped where the diffusiophoretic transport and the diffusioosmotic fluid flow balance each other, but they diffuse around the trapping position due to Brownian motion. The concentration profile of the trapped nanoparticles *C*_p_(*x*) depends on their size and zeta potential.

Diffusion determines the salt gradient in each funnel-shaped nanochannel. The boundary conditions are the fixed salt concentrations in the microchannels, that is, *C*_N_ and *C*_W_ at the narrow and wide ends of the nanochannel, respectively. So inside the nanochannel, the salt concentration is (see “Methods”)

1 $$C\left( x \right) = C_{\mathrm{N}} + \frac{{C_{\mathrm{W}} - C_{\mathrm{N}}}}{{{\mathrm{ln}}\left[ {w_{\mathrm{W}}/w_{\mathrm{N}}} \right]}}{\mathrm{ln}}\left[ {w\left( x \right)/w_{\mathrm{N}}} \right],$$

where *w*(*x*) is the local width of the nanochannel. We parametrize it as *w*(*x*) = *w*_N_ + Δ*wx*/*L*, with Δ*w* = *w*_W_ − *w*_N_, where *w*_W_ = 20 μm and *w*_N_ = 5 μm are the widths of the nanochannel at the wide and narrow ends of the nanochannel, respectively. The length of the nanochannel is *L* = 440 μm.

The salt gradient results in a diffusiophoretic particle velocity

2 $$v_{{\mathrm{ph}}}\left( x \right) = {\Gamma }_{{\mathrm{ph}}}\left( {d,\zeta } \right)\partial _x{\mathrm{ln}}C\left( x \right).$$

Here Γ_ph_ is the diffusiophoretic mobility, which depends on the particle diameter *d* and zeta potential *ζ* (for details, see “Methods”).

The diffusioosmotic fluid flow in the nanochannel is due to the local diffusioosmotic slip velocity *ν*_slip_(*x*). For a fixed solute gradient in bulk near a charged wall, the diffusioosmotic slip velocity is^26^

3 $$v_{{\mathrm{slip}}}\left( x \right) = - {\Gamma}_{{\mathrm{os}}}\left( {\zeta _{{\mathrm{ch}}}} \right)\partial _x{\mathrm{ln}}\,C\left( x \right),$$

where Γ_os_(*ζ*_ch_) is the diffusioosmotic mobility and *ζ*_ch_ is the zeta potential of the channel wall (details in “Methods”). The diffusioosmotic slip velocity causes a constant fluid flow rate *Q* due to conservation of mass, but the fluid velocity varies because of the changing channel width. We introduce a position-dependent diffusioosmotic flow velocity

4 $$v_{{\mathrm{os}}}\left( x \right) = \frac{Q}{{hw\left( x \right)}},$$

where *h* = 240 nm is the height of the nanochannel.

We then relate the diffusioosmotic flow velocity in the nanochannel *ν*_os_(*x*) to the diffusioosmotic slip velocity *ν*_slip_(*x*) by assuming that^37^

5 $$Q = w\left( x \right)hv_{{\mathrm{slip}}}\left( x \right) - \frac{{w\left( x \right)h^3\partial _xP\left( x \right)}}{{12\eta }}$$

holds locally, as the width is slowly varying compared to the length of the nanochannel (Δ*w*/*L* ≪ 1). Here ∂_*x*_*P*(*x*) is the internal pressure gradient in the fluid along the axis of the channel. It is assumed that the pressures are identical at the two ends of the nanochannel, *P*(0) = *P*(*L*). Dividing both sides in Eq. (5) with *w*(*x*) and integrating from 0 to *L* using the boundary conditions on the pressure gives *Q* = Γ_os_(*ζ*_ch_)ln(*C*_N_/*C*_W_)*h*Δw/[*L* ln(1 + Δ*w*/*w*_N_)], and, consequently, *v*_os_(*x*), see Eq. (4). Note that the flow rate only depends on the salt concentrations in the microchannels, not the specific form of the salt concentration in the nanochannel.

Particles are trapped at the position *x*_0_, where *ν*_os_(*x*_0_) + *ν*_ph_(*x*_0_) = 0. In a straight channel, the diffusioosmotic flow velocity *ν*_os_(*x*) is constant along the channel^37^, but our funnel-shaped nanochannel gives a varying fluid velocity. This results in a tighter trap.

Finally, we calculate the particle concentration in the nanochannel *C*_p_(*x*). It is related to the particle current density along the *x*-axis *J*_*x*_(*x*) as (Fick’s first law of diffusion with drift)

6 $$J_x\left( x \right) = - D_{\mathrm{p}}\partial _xC_{\mathrm{p}}\left( x \right) + \left[ {v_{{\mathrm{ph}}}\left( x \right) + v_{{\mathrm{os}}}\left( x \right)} \right]C_{\mathrm{p}}\left( x \right).$$

Here *D*_p_ = *D*_p_(*d*) is the size-dependent diffusion coefficient of particles in the nanochannel, which differs from its bulk value due to the interactions with the walls of the nanochannel (see “Methods”). The particle current *I*_p_(*x*) in the nanochannel equals the particle current density times the cross-sectional area of the nanochannel, that is, *I*_p_(*x*) = *hw*(*x*)*J*_*x*_(*x*). In steady state, *I*_p_(*x*) is constant along the nanochannel, and if particles are trapped, it vanishes. So *I*_p_(*x*) = 0, and, consequently, *J*_*x*_(*x*) = 0. For a trapping position *x*_0_ far away from both ends of the nanochannel, a solution to the particle concentration is (see Eq. (6))

7 $$C_{\mathrm{p}}\left( x \right) = C_{\mathrm{p}}\left( {x_0} \right)e^{{\int}_{x_0}^x {{\mathrm{d}}x^{\prime}\left[ {v_{{\mathrm{os}}}\left( {x^{\prime}} \right) + v_{{\mathrm{ph}}}\left( {x^{\prime}} \right)} \right]/D_{\mathrm{p}}} }.$$

Here *C*_p_(*x*_0_) = *N*/[*hw*(*x*_0_)*L*_trap_], where *N* is the number of trapped particles and $L_{{\mathrm{trap}}} = {\int}_{ - \infty }^\infty {{\mathrm{d}}x\{ {\left[ {w\left( x \right)/w\left( {x_0} \right)} \right]{\mathrm{exp}}[ {{\int}_{x_0}^x {{\mathrm{d}}x^{\prime}[ {v_{{\mathrm{os}}}\left( {x^{\prime}} \right) + v_{{\mathrm{ph}}}\left( {x^{\prime}} \right)} ]/D_{\mathrm{p}}} } ]} \}}$ is the effective length of the trap. That is, the particle distribution is normalized such that an integration over the volume of the nanochannel gives the number of trapped particles, ${\int}_{ - \infty }^\infty {{\mathrm{d}}x\;hw\left( x \right)C_{\mathrm{p}}\left( x \right) = N}$. The expression for *C*_p_(*x*) does not have a simple analytic form, but it is straightforward to solve numerically.

In Eq. (7), the diffusioosmotic flow velocity *ν*_os_(*x*) depends solely on the properties of the nanochannel, not the properties of the nanoparticle. So *ν*_os_(*x*) can be determined from the known zeta potential of the channel walls or a prior measurement (Supplementary Note 1). Both the diffusion coefficient *D*_p_ and the diffusiophoretic velocities *ν*_ph_(*x*) depend on the diameter of the nanoparticle, and *ν*_ph_(*x*) also depends on its zeta potential *ζ*. So a fit of Eq. (7) to an experimentally measured concentration profile has the particle diameter *d* and zeta potential *ζ* as the only free parameters, except for an arbitrary scale factor that converts between the particle concentration and measured intensity. In all fits of concentration profiles presented below, the particle diameters, zeta potentials, and scale factors are fitted with no constraints between data sets.

### Trapping of exosomes

We first demonstrate trapping, concentration and characterization of exosomes isolated from human blood serum of healthy donors. The stationary salinity gradients across the nanochannels are kept by a continuous flow of phosphate-buffered saline (PBS) through the microchannels. The concentrations are *C*_N_ and *C*_W_ at the narrow and wide ends of the nanochannels, respectively. Exosomes stained with the fluorescent dye DiO are introduced in the microchannel connected to the narrow ends of the nanochannels. At these ends of the nanochannels, diffusiophoresis dominates diffusioosmosis and particles migrate into the nanochannels and get trapped (Fig. 1e and Supplementary Movie 1).

After 90 s, 16 exosomes are trapped in the first nanochannel (see Fig. 1a). So, the particle concentration in the trap is ~400 times higher than the initial concentration in the microchannel (from 6 pM to 2.4 nM). Here the trap volume is defined as *V*_trap_ = *L*_trap_*hw*(*x*_0_) = 10.6 fL. From the known flow rate in the microchannel, its cross-sectional area of 150 (μm)^2^, and a particle concentration equal to 6 pM, we calculate that ~290 exosomes pass the entry of the nanochannel in 90 s. So the trapping efficiency of exosomes for a single nanochannel is 16/290 = 5.5%.

Figure 1e shows trapped exosomes at three different salinity gradients achieved by changing *C*_N_ while maintaining *C*_W_ at 10× PBS. Exosomes are trapped at physiologically relevant salinities (0.3–0.8× PBS), where the Debye length is ~1 nm (see “Methods”). The trapping positions and widths depend significantly on the gradient. For each measurement, a fit of the intensity profile gives the particle diameter and zeta potential, see Fig. 1f. Weighted averages over the three measurements at different salinity gradients (Fig. 1g) yields *d* = (78 ± 7) nm for the particle diameter and *ζ* = (−18 ± 1) mV for the zeta potential. The latter is confirmed by LDE as *ζ*_LDE_ = (−20 ± 5) mV. Size measurements with DLS were inconclusive due to the polydispersity of the sample, but the measured size agrees with the peak in the size distribution provided by the vendor (Supplementary Note 3).

### Characterizing individual liposomes

Liposomes can be made with desired sizes and zeta potentials, so they are well suited for establishing trapping in a salinity gradient and to perform characterization. We use liposomes with three different lipid compositions, that is, different POPC (1-palmitoyl-2-oleoyl-glycero-3-phosphocholine):POPG (1-palmitoyl-2-oleoyl-sn-glycero-3-phosphoglycerol) ratios that are either 3:1, 1:1, or 1:3, and, consequently, different zeta potentials (~−30, ~−36, and ~−48 mV). Liposomes were extruded through membranes with different pore sizes to produce subpopulations with different diameters (~70, ~110, and ~150 nm). Before loading the liposomes in the nanofluidic device, they were analyzed with DLS and LDE to benchmark our results.

We first measure the sizes and zeta potentials of individual liposomes. For a low concentration of liposomes in the microchannel, the filling rate of the trap is so slow that we can capture a single liposome in a nanochannel and track its stochastic motion for up to several minutes. So we load a nanochannel with a single liposome from an ensemble with a mean diameter *d*_DLS_ = (76 ± 3) nm and zeta potential *ζ*_LDE_ = (−28 ± 1) mV (Supplementary Movie 2). From microscopy images (Fig. 2a), we extract the time-dependent position of the particle in the nanochannel (Fig. 2b) and plot the *x*-coordinates (Fig. 2c).

We assume that in the long-time limit, the distribution of a single particle’s positions is identical to the spatial distribution for an ensemble of similar particles. So for each particle, we fit Eq. (7) to the histogram of its measured *x*-coordinates (black, dashed curves in Fig. 2d). For the histogram in the upper panel in Fig. 2d, the fit yields the particle diameter *d* = (72 ± 9) nm and the zeta potential *ζ* = (−30 ± 2) mV. This is consistent with the DLS and LDE measurements. Uncertainties are only ~15% and ~6%, respectively, of the measured values for a 40 s measurement. Other panels in Fig. 2d show histograms of *x*-coordinates from trajectories for two liposomes with different sizes (middle panel) and lipid compositions (lower panel). Notice how the changes in size and zeta potential alter the trapping positions *x*_0_ and the widths of the distributions. Figure 2e shows fitted values for the diameters and zeta potentials for particles from the POPC:POPG 3:1 population with *ζ*_LDE_ = (−28 ± 1) mV and diameters *d*_DLS_ = (76 ± 3) nm (red, dashed lines). The measurements on individual liposomes indicate that particle-to-particle variation can be resolved by tracking individual particles in the nanofluidic trap.

### Characterizing liposome ensembles

The trapping of exosomes demonstrates characterization of ensembles (Fig. 1). Here, we further validate the method with nine different combinations of liposome sizes and zeta potentials. We verify that results do not depend on the specific salinity gradient in the experiment, that all results are consistent with DLS and LDE measurement, and show how the trapping position *x*_0_ depends on the liposome parameters and the salinity gradient.

Figure 3a shows trapping of POPC:POPG 3:1 liposomes for seven different salinity gradients with ln(*C*_N_/*C*_W_) from −9.2 to −2.3. For ln(*C*_N_/*C*_W_) = −9.2, the first nanochannel captures 1000 liposomes in 10 s. That is ~6% of the liposomes that passed its entry, so the trapping efficiency is similar to that measured for exosomes. The 16 nanochannels (Fig. 1a) trap in total ~37% of the liposomes introduced in the microchannel (see Supplementary Note 4).

The widths of the distributions of trapped particles increase as the salinity gradient decreases because the diffusiophoretic and diffusioosmotic velocities decrease. Adjustment of the salt concentrations in the microchannels allows for moving the trapping position to physiological salinity (1× PBS, marked by the horizontal green lines in Fig. 3a), here for ln(*C*_N_/*C*_W_) between −4.6 and −3.5. Equation (7) is fitted to the intensity profile for all salinity gradients (Fig. 3b), and Fig. 3c displays the fitted values for the diameters and zeta potentials for all seven salinity gradients. No systematic dependence on the salinity gradient is observed for the fitted values and they are consistent with common weighted average values *d* = (75 ± 3) nm and *ζ* = (−30 ± 1) mV (dotted lines in Fig. 3c). Measurements identical to those in Fig. 3a–c were performed for the other eight types of liposomes. The results are summarized in Fig. 3d and are consistent with DLS and LDE measurements. For the data in Fig. 3d, the estimated net charges on the liposomes vary between ~1000*e* and ~7600*e* (ref. ^2^, Eq. 2.5.5).

The ability to discriminate particle populations based on size and zeta potential depends on the channel geometry as it modulates the velocity profile along the nanochannel, and hence the trapping position. For the present nanochannel design and experimental conditions, the trapping position is more sensitive to the zeta potential than the size. This is demonstrated by a comparison of the trapping positions for liposome samples with identical zeta potentials (Fig. 3e) or identical sizes (Fig. 3f) at fixed salinity gradients. Solid lines are the theoretically predicted trapping positions versus salt gradient, where the input parameters are the measured sizes and zeta potentials shown in Fig. 3d (trapping method). Notice in both cases the nonmonotonic dependence of the trapping position on the salt gradient. This occurs because the diffusioosmotic flow velocity is proportional to ln(*C*_N_/*C*_W_), while the diffusiophoretic velocity is proportional to ∂_*x*_ ln *C*(*x*). So, the latter depends nonlinearly on ln(*C*_N_/*C*_W_) (see Supplementary Note 5).

### Separating mixed liposome populations

For a fixed salt gradient, the distance between the trapping positions is only a few microns for the two largest particle sizes (*d* = 106 and 155 nm), much smaller than the widths of the spatial distributions of particles. The separation of trapping positions for different zeta potentials is significantly larger (see Fig. 3e, f). We therefore demonstrate separation of a liposome mixture based on surface characteristics and introduce an equal mixture of liposomes with identical sizes, but different lipid compositions, in the nanofluidic device. The liposomes are POPC:POPG 3:1 with diameters *d*_DLS_ = (73 ± 3) nm and zeta potentials *ζ*_LDE_ = (−28 ± 1) mV, and POPC:POPG 1:3 with *d*_DLS_ = (73 ± 3) nm and *ζ*_LDE_ = (−50 ± 3) mV. Figure 4a shows the separation of two liposome populations marked with different fluorophores (Supplementary Movie 3), where the separation is due to the different zeta potentials. Importantly, the separation is also clearly seen when both populations are marked with the same fluorophore (Fig. 4b). A fit to the particle distribution gives *d* = (75 ± 3) nm and *ζ* = (−30 ± 1) mV for POPC:POPG 3:1, and *d* = (69 ± 3) nm and *ζ* = (−48 ± 2) mV for POPC:POPG 1:3 (Fig. 4c). Results are consistent with DLS and LDE measurements, but the trapping method requires only a single measurement, and much smaller sample volumes and concentrations.

## Discussion

From a single measurement lasting less than minutes, we can accurately determine both the particle size and zeta potential for individual particles, ensembles of particles, or even particle mixtures. Data analysis is based on a closed set of equations and does not require any calibration to simulations. Device fabrication by injection molding, a scalable industrial process, allows for low-cost mass production of the device and integration with microfluidics for sample and cell handling.

With its combination of rapid measurements and a single-use device, the technology can be applied to on-chip concentration of dilute samples in applications where sample amounts are scarce, for example, in single-cell analysis. By tuning the trapping position to physiological salinity, biochemical reactions, for example, immunoreactions, can occur and be monitored in real time. A novel device design may also allow for surface charge characterization of smaller particles, for example, individual proteins^20^, at physiological salinity. The method is equally viable for other types of nanoparticles, and the integration of this nanofluidic method with microfluidics would, for example, allow for in-line characterization of nanoparticle liquid phases synthesis. Finally, liquid biopsy-based diagnostics can be envisaged. For example, by taking advantage of the high concentrations of particles in the trap for label-tree detection of cancerous extracellular vesicles with Raman spectroscopy^38^.

## Methods

### Sample preparation

Purified exosomes derived from human blood serum in PBS (NaCl 137 mM, KCl 2.7 mM, Na_2_PHO_4_ 10 mM, and KH_2_PO_4_ 1.8 mM) were purchased from BioCat. The size distribution has its maximum at a diameter of 70 nm (full size distribution is shown in Supplementary Fig. 4). Exosomes were incubated at 37 °C with the green lipofilic fluorophore DiO. Excess fluorophores were removed by spin column purification. Liposomes were prepared by mixing the lipids POPC, POPG, and fluorophore (Texas red and DiO were used) at appropriate ratios and were dissolved in a 9:1 tertiary butanol to water solution. Samples were freeze dried overnight and the lipid film was rehydrated in PBS while being vortexed at 50 °C. All POPC:POPG mixtures were divided into three subpopulations, which were extruded through 30, 50, and 100 nm filters, respectively.

### Nanochannel device fabrication

The nanochannel device was made by replicating a nickel master using injection molding of the cyclic olefin copolymer (COC) TOPAS 5013^39^. The nickel master was produced in a two-step ultraviolet (UV) lithography and reactive ion etching process making nanochanels and microchannels with heights of 240 nm and 5 μm, respectively. The injection molded part was sealed with a 150-μm-thick COC foil by UV-assisted thermal bonding.

### Particle trapping experiments

The device is coated for 30 min with a phospholipid POPC:POPG mixture dissolved in 70% ethanol to passivate the surface. The ratio of the uncharged POPC to the charged POPG also determines the zeta potential of the channel walls *ζ*_ch_, and, consequently, the diffusioosmotic flow^26,37^. We used lipid coatings with a surface potential of *ζ*_ch_ = (−24 ± 1) mV and *ζ*_ch_ = (−30 ± 1) mV for the exosome and liposome experiments, respectively. Characterization of the coatings is shown in Supplementary Note 1. To establish and maintain the salinity gradient in the nanochannels, PBS buffer solutions with two different concentrations were continuously flown through the microchannels by a 5 mbar pressure difference between the in- and outlets (Fig. 1a). Pressures were controlled by a Fluigent MFCS-EX pump with a stated instrumental error of ±0.3 mbar. The resulting flow rates in the microchannels are 450 pL min^−1^, and as the cross-sections of the microchannels are 150 (μm)^2^, the flow velocity is 50 μm s^−1^. Constant and reproducible residual flows of (36±15) fL min^−1^ were observed in the nanochannels with these pump settings (Supplementary Note 1). They correspond to a residual pressure across the nanochannel of 0.21 mbar, consistent with the instrumental error of the pump. The residual flows in the nanochannels were included in the data analysis by using *ν*_os_(*x*) = *Q*/[*hw*(*x*)] with the fitted value of *Q*, including the residual flow, shown in Supplementary Fig. 1b, rather than the theoretical expression for *Q* stated in and below Eq. (5). After the nanoparticles are introduced, it takes ~2 min before the salt gradient is established and a sufficient amount of nanoparticles are trapped. The solution is then changed to one with identical salinity, but without nanoparticles. The data acquisition is then performed on the trapped particles. The fluorescence image from the trapped particles was recorded with a Nikon eclipse Ti2 microscope with a Photometrics Evolve 512 electron-multiplying charge-coupled device camera. A CoolLED pE-300 Ultra LED was used as light source.

### Salt gradient in a funnel-shaped nanochannel

The salt concentration *C*(*x*) in the nanochannel depends on the salt concentrations in the two microchannels, that is, *C*_N_ at *x* = 0 and *C*_W_ at *x* = *L* (see Fig. 1). The salt current density along the *x*-axis is *J*(*x*) = −*D*∂_*x*_*C*(*x*), where *D* is the diffusion coefficient of the salt ions. The salt current *I*(*x*) in the nanochannel equals the salt current density times the cross-sectional area of the nanochannel, that is, *I*(*x*) = *hw*(*x*)*J*(*x*). In steady state, *I*(*x*) is constant along the nanochannel, that is, ∂_*x*_*I*(*x*) = 0, so ∂_*x*_[*w*(*x*)∂_*x*_*C*(*x*)] = 0. The solution for *C*(*x*) that fulfills the boundary conditions is shown in Eq. (1).

### Diffusiophoretic and diffusioosmotic mobilities

An expansion of the diffusiophoretic mobility Γ_ph_ to first order in *λ* = *λ*_DB_/(*d*/2) is ${\Gamma }_{{\mathrm{ph}}} = \frac{\varepsilon }{{2\eta }}\left( {\frac{{k_{\mathrm{B}}T}}{{Ze}}} \right)^2\left[ {u_0 + \lambda u_1} \right]$, where $\lambda _{{\mathrm{DB}}} = \sqrt {\varepsilon k_{\mathrm{B}}T/\left( {2e^2N_{\mathrm{A}}I} \right)}$ is the Debye length, *d* is the diameter of the particle, $\epsilon$ is the permittivity of the medium, *η* is the dynamic viscosity, *Z* is the valence of the solute, *e* is the elementary charge, $u_0 = 2\beta \frac{{Ze\zeta }}{{k_{\mathrm{B}}T}} - 4{\mathrm{ln}}\left( {1 - \gamma ^2} \right)$, and *u*_1_ = *F*_0_ + *βF*_1_ + Pe[*F*_2_ + *β*(*F*_3_ + *F*_5_) + *β*^2^*F*_4_]^26^. Here *N*_A_ is Avogadro’s number, *I* the ionic strength, and ${\mathrm{Pe}} = \frac{\epsilon }{{2\eta D}}\left( {\frac{{k_{\mathrm{B}}T}}{{Ze}}} \right)^2$ is the Peclet number. Furthermore, $\gamma = {\mathrm{tanh}}( {\overline \zeta /4} )$, $\overline \zeta = Ze\zeta /\left( {k_{\mathrm{B}}T} \right)$, *β* = (*D*_+_ − *D*_−_)/(*D*_+_ + *D*_−_), and *D* = 2*D*_+_*D*_−_/(*D*_+_ + *D*_−_), where *D*_+_ and *D*_−_ are the diffusion coefficient of the cations and anions of a monovalent salt, respectively. The dominant ions in PBS are sodium ions, Na^+^, and chloride ions, Cl^−^, with the diffusion constants $D_{{\mathrm{Na}}^ + } = 1330\left( {\upmu {\mathrm{m}}} \right)^2{\mathrm{s}}^{ - 1}$ and $D_{{\mathrm{Cl}}^ - } = 2030\left( {\upmu {\mathrm{m}}} \right)^2{\mathrm{s}}^{ - 1}$, respectively^30^. Thus, *β* ≃ −0.20 for NaCl. The *F*-functions depend on $\overline \zeta$ and are tabulated in Table 2 in ref. ^26^.

The diffusioosmotic mobility is ${\mathrm{\Gamma }}_{{\mathrm{os}}} = \frac{\epsilon }{\eta }\frac{{k_{\mathrm{B}}T}}{{Ze}}[ {\beta \zeta _{{\mathrm{ch}}} - 2\frac{{k_{\mathrm{B}}T}}{{Ze}}{\mathrm{ln}}\left( {1 - \gamma ^2} \right)} ]$^26^.

### Diffusion between walls

A particle diffusing between walls has a different diffusion coefficient *D*_p_ than in bulk, where the diffusion coefficient is *D*_0_ = *k*_B_*T*/(6*πηd*/2). So an expression for the diffusion coefficient of a particle in a nanochannel is required to determine the particle size and zeta potential from a concentration profile, see Eq. (7). We here use *D*_p_ = [1 − 1.004(*d*/*h*) + 0.418(*d*/*h*)^3^ + 0.21(*d*/*h*)^4^ − 0.169(*d*/*h*)^5^]*D*_0_^40^, an expansion in the ratio between the particle diameter *d* and the channel height *h* that is only strictly valid for a particle midway between two infinite walls. In Supplementary Note 2, we test experimentally the validity of the expression for our device and nanoparticles.

### DLS and LDE

DLS and LDE measurements were performed with a Zetasizer Nano Z instrument to determine the nanoparticles’ diameters and zeta potentials, respectively.

## Supplementary information

Supplementary Information

Description of Additional Supplementary Files

Supplementary Movie 1

Supplementary Movie 2

Supplementary Movie 3

## Data Availability

The data that support the findings of this study are available from the corresponding authors upon reasonable request.

## Code availability

The code used in this study is available from the corresponding authors upon reasonable request.
