# Supplementary material for: Size and surface charge characterization of nanoparticles with a salt gradient
Source: Nat Commun. 2020 May 11;11:2337. doi: 10.1038/s41467-020-15889-3 (PMC7214416; doi:10.1038/s41467-020-15889-3)
Supplement: Supplementary file 1 — Supplementary Information [file 41467_2020_15889_MOESM1_ESM.pdf]

Supplementary Information
Size and surface charge characterization of nanoparticles with a salt gradient

Rasmussen et al.

Supplementary Note 1: Flow rate measurements in nanochannels

Accurate particle characterization based on the concentration profile of trapped particles requires that the diffusioosmotic velocity is known. The diffusioosmotic flow velocity depends on the salinity gradient and the zeta potential of the channel walls ζ_{ch}. We find the flow rate, and, consequently, ζ_{ch}, following the work by Lee et *al.* [1]. That is, we measure the flow rate from the position-dependent concentration of a fluorescent dye in the nanochannel. The dye concentration ρ is kept constant at the two microchannels, which serve as reservoirs with $\rho(x=0)=\rho_N$ and $\rho(x=L)=\rho_W$.

In the funnel-shaped nanochannel, the position-dependent width is $w(x)=w_N+\Delta w x/L$. As the flow rate $Q = vhw(x)$ is constant due to the conservation of mass, the velocity is also position-dependent $v(x)=Q/[hw(x)]$. The dye current $I_{\mathrm{dye}}(x)=hw(x)J_{\mathrm{dye}}(x)$ is constant along the nanochannel in steady state, i.e., $\partial_x I_{\mathrm{dye}}(x)=0$, where $J_{\mathrm{dye}}(x) = -D\partial_x\rho(x)+v(x)\rho(x)$ due to the finite flow $v(x)$ and D is the diffusion coefficient of the dye.

The dye concentration thus fulfills

$$-Dhw(x)\partial_x\rho(x)+Q\rho(x)=\mathrm{constant} \tag{1}$$

with the boundary conditions $\rho(0)=\rho_N$ and $\rho(L)=\rho_W$. The solution is

$$\rho(x)=q_1+q_2w(x)^{\alpha}, \tag{2}$$

where

$$\alpha = \frac{Q}{Dh\Delta w/L}, \tag{3}$$

$$q_1 = \frac{\rho_N w_W^{\alpha}-\rho_W w_N^{\alpha}}{w_W^{\alpha}-w_N^{\alpha}}, \tag{4}$$

$$q_2 = \frac{\rho_W-\rho_N}{w_W^{\alpha}-w_N^{\alpha}}. \tag{5}$$

A fit of Supplementary Equation 2 to an experimental intensity profile has the flow rate Q as the only free parameter since the channel geometry, the dye concentrations in the microchannels, and the diffusion coefficient of the fluorescent dye are known. From measurements of Q versus $\ln(C_N/C_W)$, the diffusioosmotic mobility, and finally ζ_{ch}, can be extracted (see the main text). In our experiments, we used streptavidin as the fluorescent dye, which has the diffusion coefficient $D = 70\,(\mu\mathrm{m})^2\,\mathrm{s}^{-1}$ at our experimental conditions. Measurements were performed for the two lipid coatings used in the experiments, POPC:POPG 3:1 and POPC:POPG 1:1.

Supplementary Figure 1a shows a fluorescence microscope image of the fluorescent dye in POPG:POPC 3:1 coated nanochannel for an imposed salinity gradient. Intensity profiles and fits to Supplementary Equation 2 are plotted in Supplementary Figure 1b. Fitted values for the flow rates are shown in Supplementary Figure 1c together with linear fits with $Q=\frac{h}{L}\left[\Delta w/\ln\left(1+\frac{\Delta w}{w_N}\right)\right]\Gamma_{\mathrm{os}}\ln(C_N/C_W)+Q_{\mathrm{res}}$, where Q_{res} is a residual flow in the device due to a pressure difference ΔP between the channel entries. From Γ_{os}, the values of the channel zeta potentials are $\zeta = (-24\pm1)\,\mathrm{mV}$ for POPC:POPG 3:1 and $\zeta=(-30\pm1)\,\mathrm{mV}$ for POPC:POPG 1:1. The residual flows Q_{res} are $(36\pm15)\,\mathrm{fL\,min^{-1}}$ and $(29\pm13)\,\mathrm{fL\,min^{-1}}$ for POPC:POPG 3:1 and POPC:POPG 1:1 coated nanochannels, respectively. This corresponds to residual pressures ΔP equal to $(0.21\pm0.09)\,\mathrm{mbar}$ and $(0.18\pm0.08)\,\mathrm{mbar}$, respectively, which are within the instrumental error of the pressure pump ($\pm0.3\,\mathrm{mbar}$).

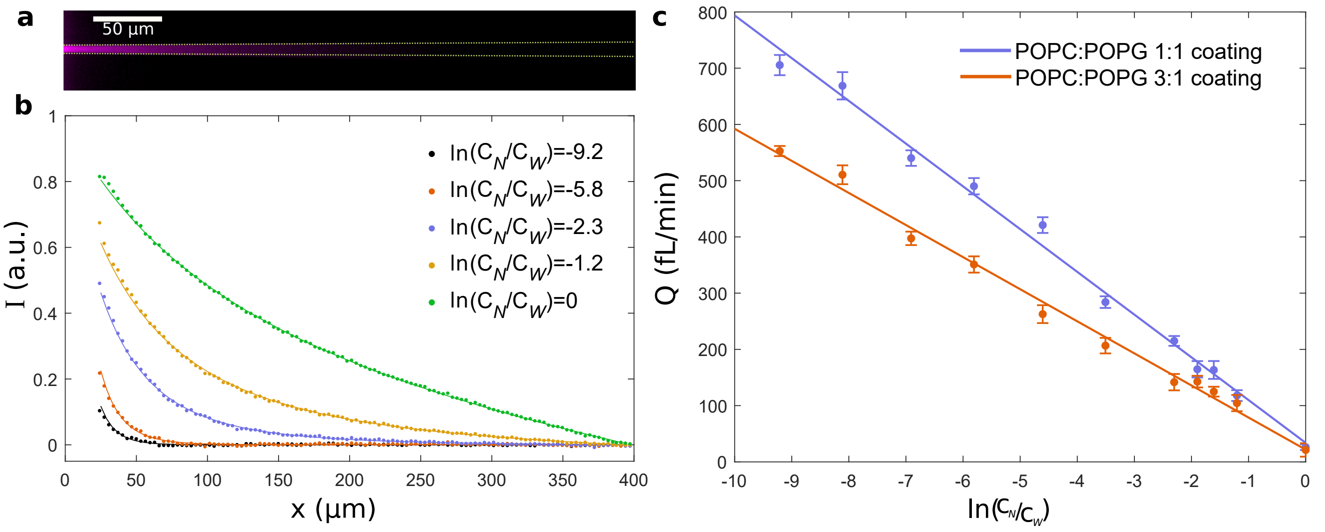

Supplementary Figure 1: Measurements of flow rates in funnel-shaped nanochannels. **a**, Fluorescence microscopy image of streptavidin in a nanochannel taken at $\ln(C_N/C_W) = -1.2$ for a POPC:POPG 3:1 coated chip. **b**, Intensity profiles and fits with Supplementary Equation 2 for channels with POPC:POPG 3:1 coating for different salinity gradients. **c**, Flow rates versus $\ln(C_N/C_W)$ for POPC:POPG 3:1 and POPC:POPG 1:1 coated nanochannels. Errorbars obtained as the standard error on the means (n=3).

Supplementary Note 2: Diffusion of particles in nanochannels

The concentration of trapped particles $C_p(x)$ depends on the particles' diffusion coefficients in the nanochannel (see eq. 7 in the main text). For a particle with diameter d in a solution with dynamic viscosity η, the diffusion coefficient in bulk is

$$D_0 = \frac{k_\mathrm{B}T}{6\pi\eta d/2}. \tag{6}$$

When a particle is placed midway between two infinite, parallel walls separated by a distance h, an expansion of the diffusion coefficient to fifth order in d/h is [2]:

$$D_p = D_0\left[1 - 1.004\left(\frac{d}{h}\right) + 0.418\left(\frac{d}{h}\right)^3 + 0.21\left(\frac{d}{h}\right)^4 - 0.169\left(\frac{d}{h}\right)^5\right]. \tag{7}$$

In our experiments, the nanochannel height is $h = 240\,\mathrm{nm}$ and particle diameters are in the range from $d = 70\,\mathrm{nm}$ to $d = 150\,\mathrm{nm}$, so D_p/D_0 is between 0.7 and 0.5. We experimentally confirm the validity of Supplementary Equation 7 for our experimental conditions by tracking the motion of individual liposomes in a funnel-shaped nanochannel in absence of a salinity gradient. Supplementary Figure 2a,b show the coordinates of a single particle undergoing Brownian motion in a nanochannel. The particle is recorded for $N = 200$ frames with a time-lapse of $\Delta t = 73.5\,\mathrm{ms}$. From the coordinates $\{(x_i, y_i)\}_{i=1}^{200}$, we calculate the displacements $\Delta x_i = x_{i+1} - x_i$ and $\Delta y_i = y_{i+1} - y_i$ for $i = 2, 3, 4, \ldots N$. Histograms of the displacements are shown in Supplementary Figure 2c,d.

The displacements in the x-direction has a mean value of $\overline{\Delta x} = (0.01 \pm 0.06)\,\mu\mathrm{m}$, where the uncertainty is the standard error on the mean. As $\overline{\Delta x}$ is within two standard errors from zero, we conclude that the measurement is consistent with the hypothesis of no drift in the x-direction. For the y-direction we find $\overline{\Delta y} = (-0.03 \pm 0.06)\,\mu\mathrm{m}$, which is also consistent with no drift. A chi-square goodness-of-fit test showed that the distribution of displacements in the x- and y-directions were consistent with normal distributions (full curves in Supplementary Figure 2c,d) as the p-values were 0.12 and 0.66, respectively.

The diffusion coefficients for the two directions were extracted from the displacements using the co-variance based estimator (CVE) [3, 4]. That is, we estimate the diffusion coefficient in the x-direction as

$$\hat{D}_x = \frac{\overline{(\Delta x_i)^2}}{2\Delta t} + \frac{\overline{\Delta x_i \Delta x_{i+1}}}{\Delta t}. \tag{8}$$

Here $\overline{\cdots}$ means average over $\Delta x_2, \Delta x_3 \Delta x_4, \ldots, \Delta x_N$, and the estimate for the positional error is

$$\hat{\sigma}^2 = R\overline{(\Delta x_i)^2} + (2R - 1)\overline{\Delta x_i \Delta x_{i+1}}, \tag{9}$$

where R is the motion blur coefficient that has the value $R = 0.11$ for our measurements, as we use an exposure time of 50 ms and a time-lapse $\Delta t = 73.5$ ms [3]. The variance of the estimate is

$$\mathrm{Var}(\hat{D}_x) = D_x^2 \left[\frac{6 + 4\varepsilon + 2\varepsilon^2}{N} + \frac{4(1+\varepsilon)^2}{N^2} \right], \tag{10}$$

where $\varepsilon = \sigma^2/(D\Delta t) - 2R$. The diffusion coefficient for the y-direction was found similarly. For a discussion of the advantage of the CVE compared to, e.g., extracting the diffusion coefficient from the mean-squared displacement (MSD), see, e.g., [3, 4].

For the trajectory shown in Supplementary Figure 2, the measured diffusion coefficients for the two directions are $\hat{D}_x = (4.6 \pm 0.8)\,(\mu\mathrm{m})^2\,\mathrm{s}^{-1}$ and $\hat{D}_y = (3.8 \pm 0.7)\,(\mu\mathrm{m})^2\,\mathrm{s}^{-1}$, respectively. As the two values are consistent with a common mean value, we take the average $\hat{D} = (\hat{D}_x + \hat{D}_y)/2 = (4.1 \pm 0.5)\,(\mu\mathrm{m})^2\,\mathrm{s}^{-1}$ as the value for the diffusion coefficient. All diffusion coefficients in Supplementary Figure 3 were obtained in an identical manner.

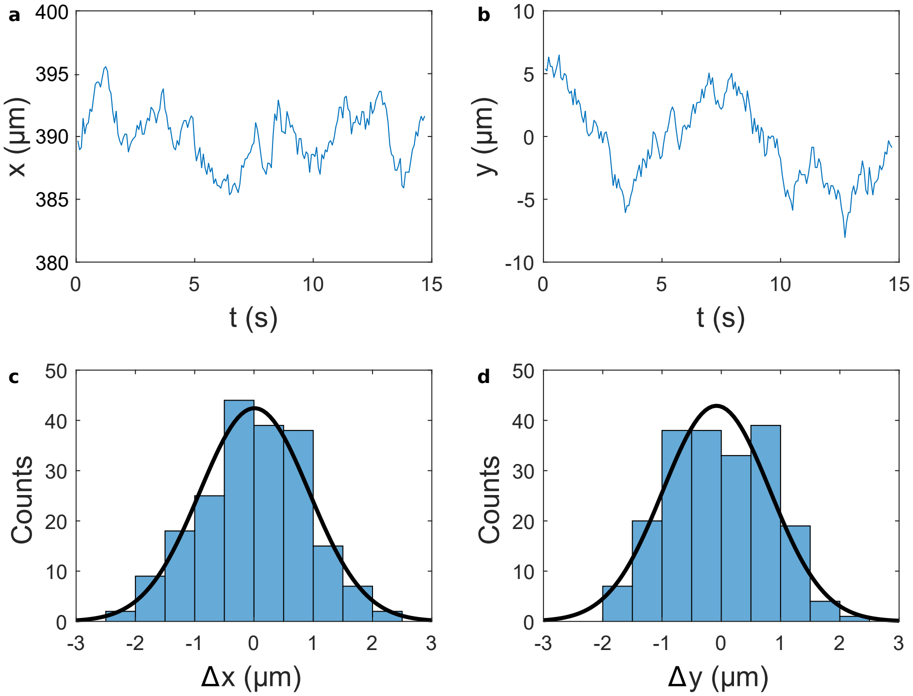

Supplementary Figure 2: Free diffusion of a particle in a nanochannel. The particle was recorded in $N = 200$ frames with a time-lapse $\Delta t = 73.5$ ms. **a,b** x and y coordinates for a particle diffusing in a nanochannel in absence of a salinity gradient. **c,d** Histograms over the displacements in the x and y-directions, respectively.

Supplementary Figure 3 shows that Supplementary Equation 7 describes the measured diffusion coefficients better than Supplementary Equation 6 for liposomes with a diameter of $d_{\mathrm{DLS}} = (76 \pm 3)$ nm. Hence we use Supplementary Equation 7 for the diffusion coefficient in the analysis of the distribution of trapped particles.

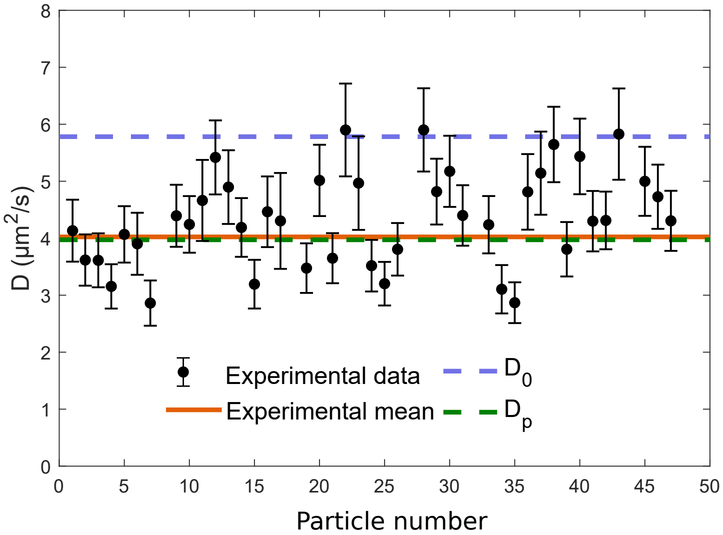

Supplementary Figure 3: Diffusion coefficients for liposomes with $d_{\mathrm{DLS}} = (76 \pm 3)\,\mathrm{nm}$ confined in a nanochannel with height $h = 240\,\mathrm{nm}$. Measurements were done in a 1xPBS solution. The diffusion coefficient for the first particle is from the trajectory shown in Supplementary Figure 2. Experimental values are compared with the theoretical predictions for particles in bulk D_0 [Supplementary Equation 6] and particles placed midway between two infinite walls D_p [Supplementary Equation 7]. Errorbars are obtained as the error on the weighted average of $\hat{D}_x$ and $\hat{D}_y$.

Supplementary Note 3: Exosome size distribution and influence on trapping

Supplementary Figure 4a shows the full size distribution of exosomes provided by the distributor (BioCat). The dashed, vertical line marks the measured diameter $d = 78\,\mathrm{nm}$ in the trapping experiment (see main text).
Supplementary Figure 4b compares the calculated concentration profiles of trapped exosomes $C_p(x)$ for the true size distribution but with a cut-off at 240 nm due to channel height restrictions (blue), and for a monodisperse sample with the diameter obtained from the trapping experiment. Only minor differences are observed, and the assumption of monodispersity used in the data analysis gives a reasonable estimate for the mean of the particle size distribution.

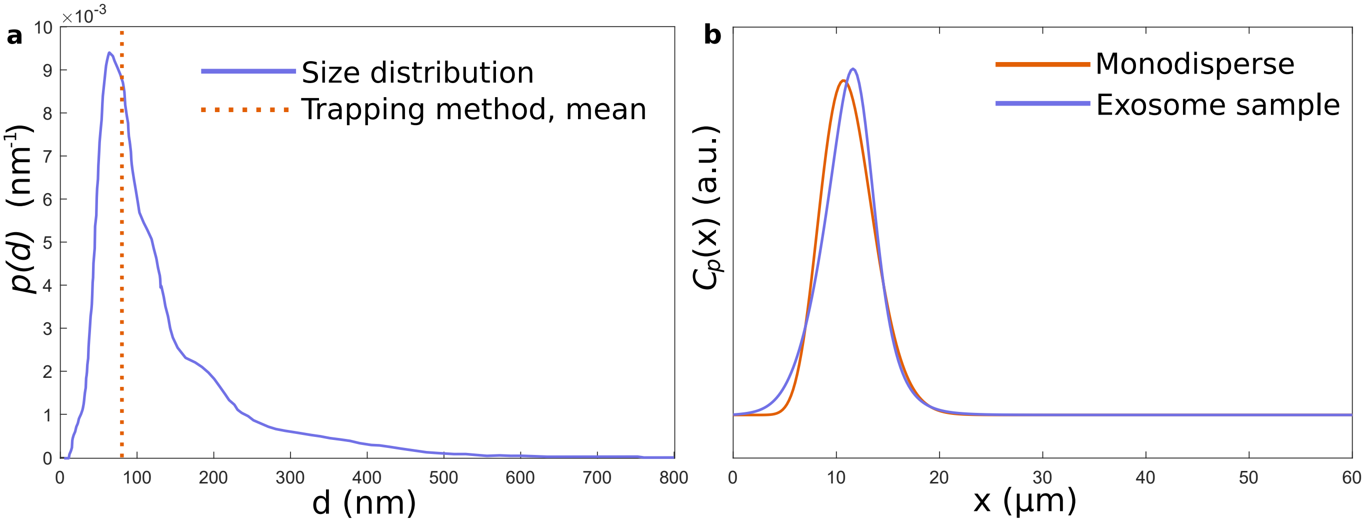

Supplementary Figure 4: Exosome size distribution and its influence on the trapping profile. **a,** Exosome size distribution. Dashed, vertical line marks the measured diameter $d = 78\,\mathrm{nm}$ in the trapping experiment. **b,** Comparison of calculated trapping profiles $C_p(x)$ for the true exosome size distribution with a cut-off at $d = 240\,\mathrm{nm}$, corresponding to the channel height, and a monodisperse sample with a diameter $d = 78\,\mathrm{nm}$ obtained from trapping experiments. Calculations were performed for $\ln(C_N/C_W) = -9.2$.

Supplementary Note 4: Trapping efficiency

We define the trapping efficiency of the device as the percentage of nanoparticles introduced in the microchannel that are trapped in the nanochannels. In the experiments nanoparticles are loaded in the upper left inlet and flow to the upper right outlet through the upper microchannel (Supplementary Figure 5). The flow rate of nanoparticles before the nanochannel array is denoted $I_{\text{micro,in}}$ (see the lower panel in Supplementary Figure 5). As the nanoparticles pass the nanochannel array, some nanoparticles enter a nanochannel and get trapped (see lower panel in Supplementary Figure 5). The flow rate of nanooparticles into the nth nanochannel is $I_{\text{trap},n}$. The nanoparticles that remain in the microchannel after the nanochannel array continue toward the outlet with a flow rate $I_{\text{micro,out}}$.

So the trapping efficiency of the device is

$$\eta_{\text{trap}} = \frac{I_{\text{micro,in}} - I_{\text{micro,out}}}{I_{\text{micro,in}}} = \frac{I_{\text{trap,total}}}{I_{\text{micro,in}}} = \frac{\sum_{n=1}^{16} I_{\text{trap},n}}{I_{\text{micro,in}}}, \tag{11}$$

where $I_{\text{trap,total}}$ is the total rate of particles entering the sixteen traps.

We find η^{trap} from two measurements. First we record the intensity of the first eleven nanochannels versus time in a single field of view for a high concentration of liposomes in the microchannel. Then we make a single-particle measurement with a low concentrations of liposomes in the microchannel, where we measure the trapping rate of the first nanochannel. All trapping efficiency measurements were performed with POPC:POPG liposomes with diameters $d_{\text{DLS}} = (76 \pm 3)$ nm in a salinity gradient defined by $\ln(C_N/C_W) = -9.2$.

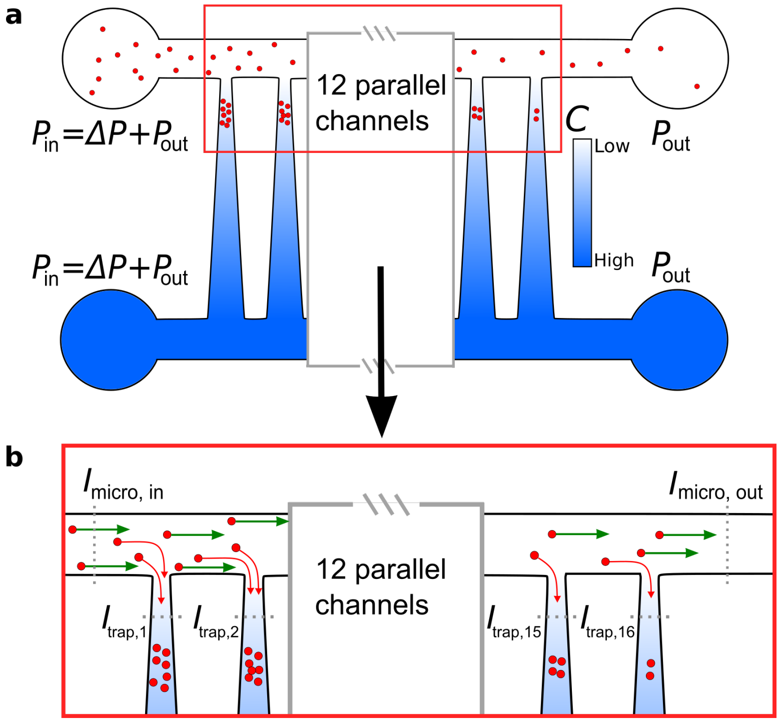

Supplementary Figure 5: Schematic of particle trapping. **a,** Particles (red dots) are loaded in the upper left inlet and flow to the upper right outlet through the upper microchannel. **a,** As the nanoparticles pass the nanochannel array, some get trapped in one of the sixteen parallel nanochannels, while the rest continues to the upper outlet.

Supplementary Figure 6a shows trapped liposomes in eleven consecutive nanochannels. The concentration of liposomes in the microchannel is $2.1 \cdot 10^{11}\,\text{mL}^{-1}$. The number of trapped liposomes in the nanochannels decreases along the flow direction as the nanochannels deplete the microchannel. We record the total fluorescence intensity of each channel versus time, see Supplementary Figure 6b. After 40 s, the intensity is proportional to time for all eleven nanochannels, which indicates a constant trapping rate in all of them. Trapping rates $I_{\text{trap},n}$ are obtained from a linear fit to data (Supplementary Figure 6b) and plotted versus channel number on a lin-log scale in Supplementary Figure 6c. The trapping rates fall on a straight line, indicating an exponential dependence

$$I_{\text{trap},n} = I_{\text{trap},1} e^{-(n-1)/\bar{n}}. \tag{12}$$

A fit gives $\bar{n} = 5.9$, and a value for $I_{\mathrm{trap},1}$ in units of intensity per time. The total trapping rate for all sixteen nanochannels is thus

$$I_{\mathrm{trap,total}} = \sum_{n=1}^{16} I_{\mathrm{trap},n} = I_{\mathrm{trap},1} \sum_{k=0}^{15} e^{-k/\bar{n}} = I_{\mathrm{trap},1} \frac{1 - e^{-16/\bar{n}}}{1 - e^{-1/\bar{n}}} \simeq 5.9\, I_{\mathrm{trap},1}. \tag{13}$$

For a low concentration of liposomes in the microchannel ($2.1 \cdot 10^9\,\mathrm{mL}^{-1}$), individual liposomes can be detected when they leave the microchannel and enter the first trap. Supplementary Figure 6d shows the number of particles trapped in the first nanochannel versus time. The number of trapped liposomes increases linearly, and the fitted trapping rate is $I_{\mathrm{trap},1} \simeq 1\,\mathrm{s}^{-1}$. So Supplementary Equation 13 gives $I_{\mathrm{trap,total}} = 5.9\,\mathrm{s}^{-1}$.

With a fluid flow velocity of $50\,\mu\mathrm{m\,s}^{-1}$ in the microchannel, a liposome concentration of $2.1 \cdot 10^9\,\mathrm{mL}^{-1}$, and a crosss-section of the microchannel equal to $150\,\mu\mathrm{m}^2$, the flow rate of liposomes in the microchannel before the nanochannel array is $I_{\mathrm{micro,in}} \simeq 16\,\mathrm{s}^{-1}$. So the first channel traps $I_{\mathrm{trap},1}/I_{\mathrm{micro,in}} \simeq 6\,\%$ of the total number of liposomes passing through the microchannel.

From the definition of the trapping efficiency in Supplementary Equation 11, $I_{\mathrm{trap,total}} \simeq 5.9\,\mathrm{s}^{-1}$, and $I_{\mathrm{micro,in}} \simeq 16\,\mathrm{s}^{-1}$, we get that the trapping efficiency for the whole device with sixteen nanochannels is

$$\eta_{\mathrm{trap}} = \frac{I_{\mathrm{trap,total}}}{I_{\mathrm{micro,in}}} \simeq 0.37. \tag{14}$$

The efficiency can be increased by decreasing the cross-section of the microchannel or by lowering the fluid flow velocity.

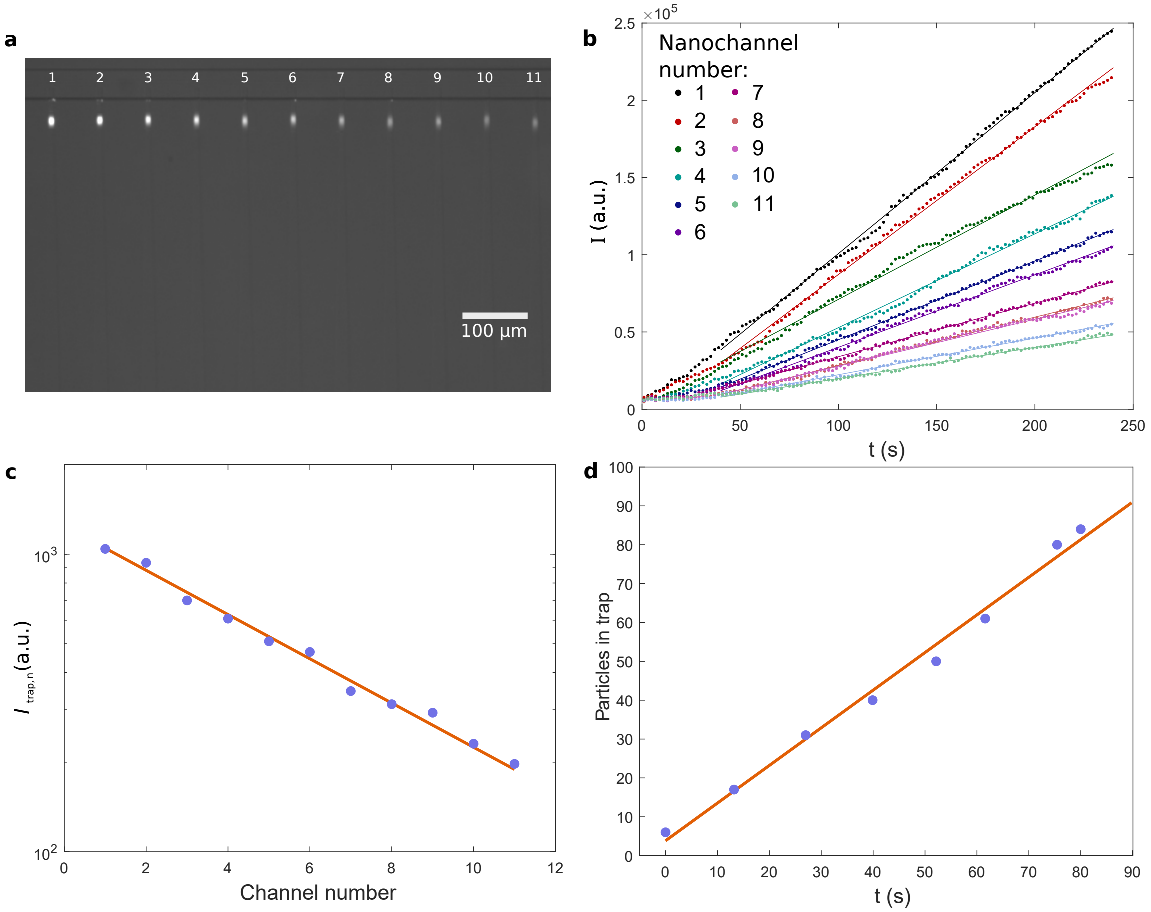

Supplementary Figure 6: Trapping efficiency. **a,** Flourescence microscopy image of liposomes trapped in parallel nanochannels. Only 11 of the 16 nanochannels are shown. Liposomes were introduced from the left in the microchannel at the top of the image. **b,** Flourescence intensity of trapped liposomes in the eleven nanochannels versus time. Linear fits give the trapping rates $I_{\mathrm{trap},n}$. **c,** Trapping rates versus nanochannel number and a fit to Supplementary Equation 12. **d,** Trapping of liposomes in the first nanochannel versus time.

Supplementary Note 5: Nonmonotonic dependence of the trapping position

The trapping position x_0 depends nonmonotonically on $\ln(C_N/C_W)$, see Fig. 3e-f in the main text. This is because the diffusioosmotic fluid velocity is proportional to $\ln(C_N/C_W)$ while the diffusiophoretic velocity depends nonlinearly on $\ln(C_N/C_W)$. The full lines in Supplementary Figure 7 show the calculated diffusiophoretic velocities $v_{\rm ph}(x)$ for a particle with a diameter $d = 75\,\rm nm$ and a zeta potential $\zeta = -30\,\rm mV$ for different values of $\ln(C_N/C_W)$. Dashed lines are the diffusioosmotic velocities $-v_{\rm os}(x)$ for the same values of $\ln(C_N/C_W)$. Trapping occurs for $v_{\rm ph}(x) + v_{\rm os}(x) = 0$ (marked with full dots). The trapping positions correspond to the values on the black curve in Fig. 3e in the main text.

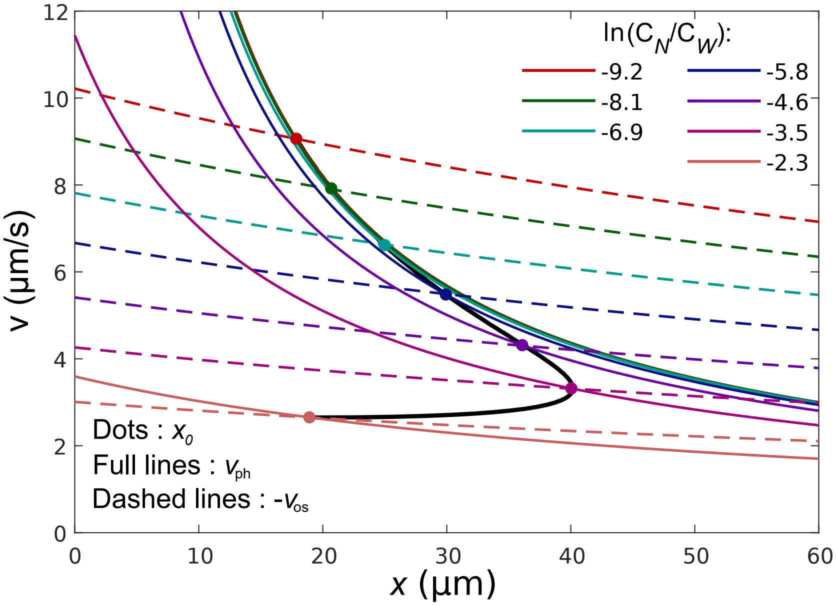

Supplementary Figure 7: Nonmonotonic dependence of the trapping postion. Trapping occurs for $v_{\rm ph}(x) + v_{\rm os}(x) = 0$, see also Fig. 1**d** in the main text.

Supplementary References

[1] Lee, C. *et al.* Osmotic flow through fully permeable nanochannels. *Phys. Rev. Lett.* **112**, 244501 (2014).

[2] Happel, H., J. & Brenner. *Low Reynolds Number Hydrodynamics* (Prentice Hall, 1965).

[3] Vestergaard, C. L., Pedersen, J. N., Mortensen, K. I. & Flyvbjerg, H. Estimation of motility parameters from trajectory data. *The European Physical Journal Special Topics* **224**, 1151–1168 (2015).

[4] Vestergaard, C. L., Blainey, P. C. & Flyvbjerg, H. Optimal estimation of diffusion coefficients from single-particle trajectories. *Phys. Rev. E* **89**, 022726 (2014).
