# Supplementary material for: Size and surface charge characterization of nanoparticles with a salt gradient
Source: Nat Commun. 2020 May 11;11:2337. doi: 10.1038/s41467-020-15889-3 (PMC7214416; doi:10.1038/s41467-020-15889-3)
Supplement: Supplementary file 2 — Description of Additional Supplementary Files [file 41467_2020_15889_MOESM2_ESM.docx]

**Description of Supplementary Files**

**File Name: Supplementary Movie 1**

**Description:** Exosomes trapped in a nanochannel due to an imposed salinity gradient with ln(Cn/Cw)= -9.2. •

**File Name: Supplementary Movie 2**

**Description:** Single liposome tracked over 40 s in the trap with ln(Cn/Cw) = -9.2. •

**File Name: Supplementary Movie 3**

**Description:** Separation of two liposome populations with similar sizes (~70 nm) in a nanochannel based on their lipid composition, POPC:POPG 3:1 (green) and POPC:POPG 1:3 (magenta), for ln(Cn/Cw) = - 9.2. Liposomes flow through the upper microchannel, but they are hardly visible due to the low light intensity required in order not to saturate the camera with the signal from the trapped liposomes.
